# Bcl-2 expression predicts radiotherapy failure in laryngeal cancer

**DOI:** 10.1038/sj.bjc.6602647

**Published:** 2005-05-31

**Authors:** P Nix, L Cawkwell, H Patmore, J Greenman, N Stafford

**Affiliations:** 1 Postgraduate Medical Institute, University of Hull in association with Hull York Medical School, Hull, UK

**Keywords:** radioresistance, apoptosis, larynx

## Abstract

Early stage laryngeal cancer can be effectively cured by radiotherapy or conservative laryngeal surgery. In the UK, radiotherapy is the preferred first line treatment. However, up to 25% of patients with T2 tumours will demonstrate locally persistent or recurrent disease at the original site, requiring salvage surgery to achieve a definitive cure. Patients experiencing treatment failure have a relatively poor prognosis. A retrospective analysis was conducted consisting of 124 patients with early stage (T1–T2, N0) laryngeal squamous cell carcinoma. In total, 62 patients who failed radiotherapy were matched for T stage, laryngeal subsite and smoking history to a group of 62 patients successfully cured by radiotherapy. Using immunohistochemistry the groups were compared for expression of apoptotic proteins: bcl-2, bcl-X_L_, bax, bak and survivin. Radioresistant laryngeal cancer was associated with bcl-2 (*P*<0.001) and bcl-X_L_ (*P*=0.005) expression and loss of bax expression (*P*=0.012) in pretreatment biopsies. Bcl-2 has an accuracy of 71% in predicting radiotherapy outcome. The association between expression of bcl-2, bcl-X_L_ and bax with radioresistant cancer suggests a potential mechanism by which cancer cells avoid the destructive effects of radiotherapy. Predicting radioresistance, using bcl-2, would allow the clinician to recommend conservative laryngeal surgery as an alternative first line treatment to radiotherapy.

Head and neck cancers are the sixth most prevalent cancers in the world with a global incidence of 700 000 cases per year (Parkin *et al*, 2001). They constitute a heterogeneous group of cancers arising from the upper aerodigestive tract, paranasal sinuses, salivary and thyroid glands. In order to avoid tumour heterogeneity we have investigated one region, the larynx. This is the largest head and neck region in the United Kingdom affected by cancer, with the vast majority of tumours being squamous cell in origin. Cancer in this region has important functional and psychological consequences for the patient, in particular with regard to communication and eating.

Radiotherapy not only cures most early stage, T1 and T2, laryngeal cancers but it also preserves laryngeal function. Unfortunately, treatment failures do occur; between 5 and 10% of stage I and up to 25% of stage II laryngeal cancers are resistant to radiotherapy (Fernberg *et al*, 1989; Klintenberg *et al*, 1996; Johansen *et al*, 2002). Patients then require salvage surgery, typically involving removal of the larynx, in order to achieve tumour control. The delay in diagnosis following failed radiotherapy results in tumour progression, which impacts significantly on patient survival. Salvage surgery is also associated with increased failure and complication rates due to operating in a previously irradiated field (McLaughlin *et al*, 1996).

When a patient presents with head and neck cancer, subsequent management and treatment is based largely upon the TNM staging of the tumour. While this system is adequate in predicting group responses to treatment, there is significant variability between individual patients within any particular stage. Although there have been a number of laboratory studies on radioresistance in early laryngeal cancer, at present there are no predictive molecular markers in routine use (Smith and Haffty 1999; Yoo *et al*, 2000; Cho *et al*, 2004; Nix *et al*, 2004a, 2004b).

Radiotherapy results in DNA damage, the most lethal form of which is a double-strand break (Willers *et al*, 2004). If the cell is unable to repair such damage, a type of programmed cell death is initiated termed apoptosis (Kerr *et al*, 1994). The Bcl-2 family of proteins play an important part in a cell's ability to undergo apoptosis following radiotherapy damage (Gudkov and Komarova 2003). The antiapoptotic members of the family, bcl-2 and bcl-X_L_, and the proapoptotic member bax are reportedly expressed in 15, 74 and 81%, respectively, of laryngeal biopsy samples from advanced laryngeal tumours (Trask *et al*, 2002). Bcl-2 overexpression, in relation to radiotherapy treatment failures, has also been reported in cervical (Mukherjee *et al*, 2001), prostate, bladder (Pollack *et al*, 1997), rectal (Schwandner *et al*, 2000) and non-small-cell-lung cancer (Hwang *et al*, 2001). In a study of eight cases of radioresistant laryngeal cancer and 13 cases of radiosensitive cancer, the expression of key proteins involved in DNA damage recognition (p53), cell cycle arrest (ATM, p16 and p21/WAF1) and apoptosis (bcl-2 and bax) was studied. Bcl-2 was found to be significantly associated with radioresistant tumours (*P*=0.03) (Condon *et al*, 2002).

Antiapoptotic members of the Bcl-2 family of proteins, such as bcl-2 and bcl-X_L_, prevent cytochrome *c* release from the mitochondrial membrane. Proapoptotic members, such as bax and bak, cause the release of cytochrome *c* (Wei *et al*, 2001). Once cytochrome *c* is released into the cytosol, it activates procaspase 9 resulting in an enzymatic cascade that eventually activates the effector caspases 3, 6 and 7. Caspases are an important family of proteases responsible for effecting programmed cell death. They are activated in response to excessive cell stress such as radiotherapy induced DNA damage (Mow *et al*, 2001). The Bcl-2 family also controls the release of apoptosis inducing factor (AIF) and EndoG nuclease from the mitochondria (Susin *et al*, 1999). This control is again partly dependent upon relative concentrations of the family members. AIF and EndoG nuclease migrate into the cell nucleus and cause chromatin condensation and DNA fragmentation. This apoptotic pathway is independent of cytochrome *c* and occurs following radiotherapy induced cell damage (Ravi and Bedi, 2002). Survivin is a member of the inhibitors of the apoptosis family. It functions downstream of the Bcl-2 family by inhibiting the actions of caspases 3 and 7 (Tamm *et al*, 1998). Overexpression of survivin has been associated with radioresistance in colorectal cell lines (Rodel *et al*, 2003).

On the basis of these observations we have investigated the relationship between members of the Bcl-2 family (bcl-2, bcl-X_L_, bax, bak) and survivin with radioresistant laryngeal cancer.

## MATERIALS AND METHODS

### Study population and definitions

Local Research Ethics Committees' approval was obtained for the study using archival biopsy material. Two groups of patients with laryngeal carcinoma, treated with curative intent by 55–60Gy of radiotherapy in 20–25 fractions, were retrospectively identified from databases held in ENT Departments in England. Following completion of single modality radiotherapy, all patients were followed up in a Head and Neck Oncology Clinic. Patients were reviewed on a monthly basis during the first year, a bimonthly basis in the second year and every 3 months during the third year. One group consisted of patients with radioresistant laryngeal tumours and the other group with radiosensitive tumours.

The criteria for a radioresistant tumour was the following:

1. Radiotherapy had to be given as a single modality treatment with curative intent for a biopsy proven squamous cell carcinoma of the larynx.
2. Biopsy proven recurrent squamous cell carcinoma occurring at the original anatomical site, within 12 months of finishing a course of radiotherapy.

The criteria for a radiosensitive tumour was the following:

1. Biopsy proven squamous cell carcinoma of the larynx resulted in single modality treatment with radiotherapy.
2. Post-treatment, patients had a minimum follow up of 3 years following completion of radiotherapy with no evidence of recurrence at the original site of the tumour.

In order to reduce confounding variables, the two groups were matched with regard to laryngeal T stage, glottic or supraglottic cancer subsite and smoking history. Each group consisted of 62 patients comprising 44 T1- and 18 T2-staged laryngeal squamous cell carcinomas (Table 1). Clinically all tumours were N0 and M0 at the time of treatment, according to the American Joint Committee on Cancer classification 2002 (Greene and Sobin, 2002). In total, 56 tumours occurred in the glottic and six in the supraglottic subsites in each group.

### Procedure

Tissue sections were taken from pretreatment archival tissue blocks and immunohistochemical techniques were used to detect the apoptotic markers bcl-2, bcl-X_L_, bax, bak and survivin. The avidin biotin method, as previously described (Cawkwell *et al*, 1999), allowed the immunohistochemical detection of the monoclonal antibodies: anti-bcl-2 at a dilution of 1 : 50 (Neomarkers, Fremont, USA: Ab-1 clone 100/D5), anti-bcl-X_L_ at a dilution of 1 : 75 (Neomarkers: Ab-2 clone 7D9), anti-bax at a dilution of 1 in 75 (Neomarkers: Ab-5 clone 2C8), anti-survivin at a dilution of 1 in 50 (Santa Cruz Biotechnology, USA FL-142) and the polyclonal antibody anti-bak at a dilution of 1 : 50 (Neomarkers Ab-2). The negative control for each marker was to omit the primary antibody. Immunohistochemistry was performed in batches of 50 with equal radioresistant and radiosensitive samples in each batch in order to minimise any influence of assay run on variability.

Two investigators performed marker scoring independently, with the radiotherapy treatment outcome blinded to the scorers. If consensus agreement could not be reached, a third opinion was taken. In order to reduce sampling error, the whole pretreatment laryngeal tumour biopsy was assessed for antibody staining. Intensity of marker staining was not used as a method for scoring the tissue sections because the degree of antigen masking is dependent upon the fixative used, its degree of penetration and the fixation time. Such factors may vary between clinical samples and as a consequence are difficult to control for. Laryngeal tumour sections were regarded as positive only if more than 5% of the tumour cells displayed the staining when viewed by light microscopy at × 200 magnification. Sections with 5% or less of the tumour staining were considered negative. This simple positive or negative scoring system used to interpret the antibody staining patterns has previously been reported and results in high degree of interobserver agreement (Mighell *et al*, 1998). In this study, the two independent assessors had complete agreement in 100% of the bak, bad and survivin cases. For bcl-2 and bcl-X_L_, the two assessors had complete agreement in 90 and 87% of the cases, respectively, and consensus agreement was achieved for all cases.

### Statistical analysis

*χ*^2^ statistical analysis using two by two contingency tables with one degree of freedom was used to calculate significance. All *P*-values quoted are for two-sided significance, between the radioresistant and radiosensitive groups with values less than 0.05 being considered significant. Multiple regression analysis was performed using SPSS v11.5 (SPSS Inc.). Marker accuracy, sensitivity and specificity were calculated as previously reported (Greenhalgh, 1997).

## RESULTS

The radioresistant group consisted of 62 patients with laryngeal squamous cell carcinoma (Table 1). The average age of the patient at diagnosis was 64 with a range of 45–87 years. The mean time to tumour recurrence was 6.2 months with a range of 2–12 months. The average age of the radiosensitive group was 62 with a range of 40–87 years. All radiosensitive tumours had been followed up for at least 3 years with no evidence of a recurrent tumour. There was no significant difference in tumour differentiation (*P*=0.543) or gender (*P*=0.610) between the two groups (Table 1).

Bak staining was localised to the cytoplasm of the tumour and was present in all radioresistant and radiosensitive tumours. Survivin expression was also present in all radioresistant and radiosensitive tumours, displaying a cytoplasmic and nuclear distribution (Table 2).

Bcl-2 and bcl-X_L_ staining was localised to the tumour cell cytoplasm. Intense staining was also evident in stromal lymphocytes. Bax staining was diffusely present in the cytoplasm of tumour cells and localised in a granular pattern in the cell nucleus. Again this staining pattern was also present in stromal lymphocytes.

In total, 53% of radioresistant tumours expressed bcl-2 compared with 11% in the radiosensitive group (*P*<0.001). Bcl-X_L_ was expressed by 91% of the radioresistant tumours compared with 73% of the radiosensitive tumours (*P*=0.005). In total, 43% of radioresistant tumours expressed bax compared with 66% in the radiosensitive group (*P*=0.012). Radioresistant tumours were associated with expression of the antiapoptotic markers bcl-2 and bcl-X_L_ and underexpression of the proapoptotic marker bax (Table 2).

The coexpression of the apoptotic markers, bcl-2, bcl-X_L_ and bax in radioresistant laryngeal cancer, is displayed in Table 3. The main findings are that 50% of the radioresistant samples expressed both bcl-2 and bcl-X_L_, while only 5% of the tumours were negative for both markers. Bcl-2 expression and loss of bax expression was seen in 31% of the radioresistant tumours. Bcl-X_L_ expression and loss of bax expression was seen in 50% of the radioresistant tumours.

Multivariate regression analysis using treatment failure as the dependent factor and bcl-2, bcl-X_L_, bax, bak, survivin, tumour differentiation as covariants demonstrated that bcl-2 (*P*<0.001), bcl-X_L_ (*P*=0.007) and bax (*P*=0.014) were independent variables.

In this series, bcl-2 expression has an accuracy of 71% in predicting the outcome of radiotherapy with a sensitivity of 53% and a specificity of 89% (Table 4). Bcl-2 has been chosen as a predictor of radioresistance in preference to bcl-X_L_ or bax due to its low false positive rate of 11%.

## DISCUSSION

At present, a clinical equipoise exists in the management of early stage laryngeal cancer. Radiotherapy and conservative endolaryngeal surgery are currently used to obtain a cure, with no published randomised controlled trials to suggest the most effective modality (Dey *et al*, 2002). Treatment choice usually depends upon local available expertise and the physician's preference for treatment options. Using markers that can predict the radioresistance of a tumour would enable the TNM staging system to be refined and allow tailored cancer treatments to be devised for each patient.

Each head and neck region has its own TNM staging system (Greene and Sobin, 2002), such that a T1 tumour in one region is not comparable to similar staged tumours in other regions with regard to treatment regimes and treatment failure rates (BAO-HNS, 2002). Investigating radioresistance using heterogeneous tumour groups has led to conflicting published results. To date, cellular and molecular markers of radioresistance in head and neck cancer have failed to improve the accuracy of the TNM system (Nix *et al*, 2004a). In order to address this issue we have assembled a large homogeneous series of radioresistant laryngeal cancers. As there is no universal definition of radioresistant cancer, we have devised a strict definition. By stipulating that recurrences have to occur within 12 months of finishing radiotherapy, second primary tumours are very unlikely to be counted as an erroneous radiotherapy recurrence. Second primary cancers are frequent in head and neck regions, occurring at a rate of 7% per year following the index case (Holland *et al*, 2002). In addition, we have only used pretreatment archival biopsy material in the study. This is to avoid any effects that radiotherapy may have on the markers under investigation. As this is a nonrandomised retrospective review, we have tried to minimise confounding variables by matching the groups as closely as possible for TNM stage, laryngeal subsite, smoking history, gender and radiotherapy dose and schedule.

We have used a simple method to evaluate the immunohistochemical staining pattern, as opposed to semiquantitative scales based on intensity; this is because intensity is partly dependent upon the formalin fixation procedure (Fisher *et al*, 1994).

This study demonstrates that there is a differential expression of the Bcl-2 family members between radioresistant and radiosensitive laryngeal cancer. The majority of early stage laryngeal cancers are radiosensitive. As a consequence, our radiosensitive tumours should be similar to reported series that do not differentiate radioresistant and radiosensitive tumours. The overexpression of bax (66%) and the under expression of bcl-2 (11%) and bcl-X_L_ (73%), in our radiosensitive series, are similar to reported results in other laryngeal series looking at apoptotic marker expression (Trask *et al*, 2002). In contrast, there is a statistically significant difference in bcl-2 (53%) and bcl-X_L_ (91%) expression and bax (43%) underexpression in our radioresistant group compared to the radiosensitive group.

The Bcl-2 family members all possess at least one of four conserved functional motifs, which allows hetero- and homo-dimerisation between family members. When dimerisation occurs between pro- and antiapoptotic members, their action is effectively neutralised (Wei *et al*, 2001). As a consequence, the relative concentrations of the family members are one way that the apoptotic machinery is regulated (Ravi and Bedi, 2002). This may explain the differential distribution of markers between the two groups. In this study, the Bcl-2 family members that oppose apoptosis are significantly associated with the radioresistant tumour specimens. The overexpression of bcl-2 and bcl-X_L_ proteins by the tumour cells may create a block to radiotherapy-induced apoptosis. As a consequence, the tumour becomes relatively radioresistant. Also by downregulating the level of the proapoptotic Bcl-2 family member, bax, radiotherapy-induced apoptosis will be further inhibited.

As early stage laryngeal tumours can be effectively cured by either conservative laryngeal surgery or radiotherapy (Ton-Van *et al*, 1991), predicting which tumours are radioresistant means that these patients can be offered a surgical option initially. In the UK approximately 2300 patients develop laryngeal cancer (BAO-HNS, 2002), of which 1150 will be treated with radiotherapy; and of these up to 287 (25%) will be radioresistant. At present, the clinician cannot predict any of the above radiotherapy treatment failures. However, using bcl-2, we can predict 152 (53%) of the failures and offer these patients conservative laryngeal surgery instead of radiotherapy. Conservative laryngeal surgery is an established technique, widely used in the USA and Europe as a first-line treatment for early stage laryngeal cancer (BAO-HNS, 2002); hence, such patients will benefit from improved survival and quality of life as their larynx will be preserved and they will not have to receive unnecessary radiotherapy. Equally, there will be no detrimental effect to the 11% of patients with a false positive bcl-2 result, who will be offered conservative laryngeal surgery instead of radiotherapy.

By identifying the mechanism that tumour cells develop, radioresistance novel treatment therapies may be developed. Antisense oligonucleotides targeting bcl-2 have been used to reduce the expression of bcl-2 in phase 1 clinical trials with lymphomas (Waters *et al*, 2000). This strategy could be used to reduce the expression of bcl-2 or bcl-X_L_ in cancers that are predicted to be radioresistant. This therapy may then lead to a more radio-responsive tumour. Such a strategy is highly likely to be beneficial for bcl-2 positive advanced stage laryngeal tumours that are currently managed with combination surgery and radiotherapy or combined chemo-radiotherapy.

By studying a homogeneous group of head and neck cancer, using a strict definition of radioresistance to avoid confounding factors, we have demonstrated that expression of antiapoptotic members, bcl-2 and bcl-X_L_, and underexpression of proapoptotic marker, bax, are associated with radioresistance. By only studying pretreatment biopsy samples, bcl-2 has a 71% accuracy in predicting patient response to radiotherapy. These observations now require verification in larger-scale prospective trials. Case–control studies rely on retrospective records and there may be confounding study factors, which can be more closely controlled in a prospective clinical trial. Ideally, a randomised controlled trial between radiotherapy and conservative endolaryngeal surgery would also address the issue that bcl-2 predicts radioresistance as opposed to just being a marker of poor outcome. If verified, Bcl-2 positive patients could be offered an established and equally effective alternative treatment to radiotherapy, resulting in improved patient survival and quality of life.

## Figures and Tables

**Table 1:** Radioresistant and radiosensitive patient profiles

	**Radioresistant patients**	**Radiosensitive patients**
	**(*N*=62)**	**(*N*=62)**
*T1 staged tumour*		
Glottic	42	42
Supraglottic	2	2
		
*T2 staged tumour*		
Glottic	14	14
Supraglottic	4	4
		
*Histological grade*		
Well	19	20
Moderate	35	30
Poor	8	12
		
*Gender*		
Female	8	10
Male	54	52
		
Average age (years)	64	62

**Table 2:** Expression pattern of bcl-2, bcl-XL, bax, bak and survivin

**Marker**	**Radioresistant tumour numbers (62)**	**Radiosensitive tumour numbers (62)**	***P* value^a^**
bcl-2 positive	33 (53%)	7 (11%)	*P*⩽0.001
bcl-2 negative	29 (47%)	55 (89%)	
			
bcl-X_L_ positive	57 (91%)	45 (73%)	*P*=0.005
bcl-X_L_ negative	5 (9%)	17 (27%)	
			
bax positive	27 (43%)	41 (66%)	*P*=0.012
bax negative	35 (57%)	21 (34%)	
			
bak positive	62 (100%)	62 (100%)	
bak negative	0	0	
			
survivin positive^b^	37 (100%)	37 (100%)	
survivin negative	0	0	

a *χ*^2^ test, two-sided significance.

b *n*=37.

**Table 3:** Marker expression in 62 radioresistant laryngeal cancers

	**Bcl-X_L_**	
	**Negative stain**	**Positive stain**
Bcl-2 negative stain	3 (5%)^a^	26 (42%)
Bcl-2 positive stain	2 (3%)	31 (50%)
		
	**Bax**	
	**Negative stain**	**Positive stain**
Bcl-2 negative stain	16 (26%)	13 (21%)
Bcl-2 positive stain	19 (31%)	14 (23%)
		
	**Bax**	
	**Negative stain**	**Positive stain**
Bcl-X_L_ negative stain	4 (6%)	1 (2%)
Bcl-X_L_ positive stain	31 (50%)	26 (42%)

a Percentages quoted are based on the cohort of 62 radioresistant tumours.

**Table 4:** Predictive value of bcl-2 as a marker of radiotherapy outcome in 124 patients

	**Final outcome of therapy**	
**Bcl-2 staining**	**Tumour recurrence=62**	**Tumour free=62**
Positive=40	True +ve=33	False +ve=7
Negative=64	False −ve=29	True +ve=55
		
Sensitivity	53%	
Specificity	89%	
Positive predictive value	83%	
Negative predictive value	65%	
Accuracy	71%	
